# Reviewed article: Araújo ES, Batista JFC, Santos YIN, Almeida-Santos MA. Temporal trend and factors associated with physical disability due to leprosyl: a time series study, Brazil, 2014-2023. Epidemiol Serv Saude. 2026:35;e20240899.

**DOI:** 10.1590/S2237-96222026v35e20240899.a

**Published:** 2026-05-01

**Authors:** Mariana Del Grossi Moura

**Affiliations:** 1 Universidade de Sorocaba, Sorocaba, SP, Brazil Universidade de Sorocaba Sorocaba SP Brazil

## First round comments

Agradeço a submissão do manuscrito à RESS. Após análise, identifico a necessidade de uma revisão substancial, especialmente em relação à estrutura metodológica, à padronização e formatação das tabelas, bem como à adequação das citações e referências ao estilo Vancouver. Além disso, a discussão precisa de melhorias para tornar a interpretação dos achados mais clara e objetiva.

Alguns pontos importantes:

- O título do manuscrito pode ser reformulado para deixar mais evidente o tipo de estudo realizado, além disso, as palavras-chave devem ser ajustadas para refletir com mais precisão o delineamento do estudo. Para isso, é fundamental que os autores utilizem os Descritores em Ciências da Saúde (DeCS), seguindo as normas estabelecidas pela revista;

- Recomendo fortemente que o manuscrito seja revisado à luz do checklist RECORD, que orienta a estruturação de estudos observacionais com dados de saúde coletados rotineiramente. Seguir essas diretrizes ajudará a assegurar um relato mais completo, estruturado e transparente de todas as etapas do estudo;

- Na seção de resultados e discussão, há repetição excessiva de números no texto, além de alguns trechos redundantes. As tabelas devem ser revisadas para garantir conformidade com o padrão editorial da revista. O primeiro parágrafo da discussão poderia ser reformulado para apresentar um resumo mais objetivo dos principais achados. Além disso, sugere-se aprofundar a relevância prática dos resultados, destacando suas implicações no contexto da saúde pública e possíveis aplicações;

- Todas as citações e referências devem ser revisadas e formatadas conforme as regras do estilo Vancouver, garantindo padronização e conformidade com as diretrizes da revista. Por fim, sugiro a verificação de similaridades no conteúdo, garantindo a originalidade do manuscrito e sua adequação ao padrão editorial da revista.

Reitero a importância dessas adequações para que o manuscrito possa avançar no processo editorial. Aguardamos a versão revisada e nos colocamos à disposição para quaisquer esclarecimentos adicionais.

Recommendation: Major revision

## Second round comments

Agradecemos a submissão do manuscrito RESS-2024-0899.R1, intitulado “Fatores associados e tendência temporal do grau de incapacidade física da hanseníase no Brasil: estudo transversal e ecológico, 2014-2023”, à Revista Epidemiologia e Serviços de Saúde: revista do SUS (RESS).

Após a revisão do seu artigo, identificamos a necessidade de uma revisão menor, a fim de adequar o manuscrito aos padrões editoriais da revista e viabilizar sua publicação.

Orientações para ajuste:

• Resumo (objetivo): Ajustar a concordância verbal para “foi calculada a taxa”. Substituir “/1 milhão” por “por 1 milhão de habitantes”, em conformidade com a terminologia padronizada em estudos epidemiológicos.

Reforçamos que, com essa reformulação, o resumo deve ser ajustado para não ultrapassar o limite de 250 palavras, conforme as normas da RESS.

• Resultados: Revisar a frase “A população final foi de 228.577”, presente no resumo e no texto completo. A expressão é genérica e pode gerar ambiguidade.

Recomendamos especificar do que se trata, utilizando expressões como “casos analisados”, “registros incluídos” ou “casos confirmados de hanseníase”.

No resumo, sugerimos a seguinte formulação, mais direta e informativa: “Foram analisados 228.577 casos confirmados de hanseníase no Brasil, entre 2014 e 2023.”

• Disponibilidade dos dados: Conforme a política editorial da RESS, é necessário indicar, na seção “Disponibilidade dos dados”, o repositório onde os dados analisados estão depositados, como SciELO Data, OSF ou similares, preferencialmente com identificador persistente (DOI).

A referência completa do banco de dados deve ser incluída na seção de “Referências” e citada no corpo do texto, preferencialmente na seção de “Métodos”.

Abaixo, reproduzimos a diretriz da RESS sobre esse item:

“Os dados de pesquisa (bancos de dados, códigos, métodos e outros materiais utilizados e resultantes da pesquisa) idealmente devem ser depositados em repositórios de dados confiáveis como o repositório SciELO Data (lista disponível em https://wp.scielo.org/wp-content/uploads/Lista-de-Repositorios-Recomendados_pt.pdf), preferencialmente com um identificador persistente como o digital object identifier (DOI). O acesso a tais dados com o link para o repositório deverá ser informado na seção ‘Disponibilidade de dados’. Deve haver citação dos dados no texto, preferencialmente nos ‘Métodos’, e a referência completa deve ser incluída na seção de ‘Referências’, seguindo exemplo:”

Andrade M. Estudo de genes em ratos albinos na América Latina. OSF [dataset], 2018, ASM0000v1. http://dx.doi.org/10.1590/0123-45620187214

Aguardamos a nova versão do manuscrito acompanhada da carta-resposta ponto a ponto, com destaque para as modificações realizadas.

Recommendation: Minor revision

## Third round comments

Agradecemos a submissão do manuscrito RESS-2024-0899.R2, intitulado “Fatores associados e tendência temporal da incapacidade física da hanseníase no Brasil: estudo transversal, 2014-2023”, à Revista Epidemiologia e Serviços de Saúde: revista do SUS (RESS).

Após a revisão do seu artigo, identificamos a necessidade de uma revisão menor, a fim de adequar o manuscrito aos padrões editoriais da revista e viabilizar sua publicação.

Orientações para ajuste:

- Padronizar a redação do objetivo do estudo no resumo e no corpo do texto (introdução), mantendo a mesma formulação em ambos.

-Resumo: O trecho “A proporção de casos novos com grau 2 de incapacidade aumentou (VPA 6,0; IC95% 5,1; 6,9)” ainda sugere uma interpretação direta (“aumentou”), o que não é recomendado. Sugestão: A variação percentual anual (VPA) foi de 6,0 (IC95% 5,1; 6,9) para os casos novos com grau 2 de incapacidade no Brasil.

- Resultados (texto principal): Ainda há expressões interpretativas nos resultados textuais que deveriam ser substituídas por linguagem neutra.

- O link informado na seção “Disponibilidade de dados” (https://doi.org/10.48331/scielodata.UOZXWF) resulta em “página não encontrada”. Solicitamos que verifiquem se o depósito dos dados foi corretamente concluído e atualizem o endereço com um link funcional e acessível ao público.

Aguardamos a nova versão do manuscrito acompanhada da carta-resposta ponto a ponto, com destaque para as modificações realizadas.

Recommendation: Minor revision

## Fouth round comments

Agradecemos o envio da nova versão do manuscrito RESS-2024-0899.R3, intitulado “Fatores associados e tendência temporal da incapacidade física da hanseníase no Brasil: estudo transversal, 2014-2023”, à Revista Epidemiologia e Serviços de Saúde: revista do SUS (RESS).

Verificamos que o link informado na seção “Disponibilidade de dados” (https://doi.org/10.48331/scielodata.UOZXWF) continua resultando em “página não encontrada”. Solicitamos, portanto, que confirmem se o depósito foi devidamente finalizado e atualizem o manuscrito com um link funcional e acessível ao público.

Além disso, destacamos que a carta-resposta ponto a ponto não foi enviada, o que inviabiliza a adequada verificação das modificações realizadas. Reforçamos a necessidade de incluir esse documento na próxima submissão.

Recommendation: Minor revision

## Fifth round comments

Agradecemos a submissão do manuscrito RESS-2024-0899, intitulado “Fatores associados e tendência temporal da incapacidade física da hanseníase no Brasil: estudo transversal, 2014-2023”, à Revista Epidemiologia e Serviços de Saúde: revista do SUS (RESS).

Após a revisão da nova versão do seu artigo, identificamos a necessidade de ajustes pontuais para adequação aos padrões editoriais da RESS e viabilização de sua publicação.

Gostaria de esclarecer que a informação obtida junto à secretaria - de que o DOI só será liberado após a publicação do artigo - refere-se exclusivamente ao identificador do artigo, e não ao DOI do conjunto de dados. São processos distintos:

• DOI do artigo: gerado e ativado somente após a publicação final do manuscrito.

• DOI do conjunto de dados: deve ser criado e funcional antes da submissão da versão final, permitindo o acesso imediato aos dados.

Conforme a política editorial da RESS, solicitamos que vocês:

1. Depositem os dados utilizados na análise em um repositório confiável (por exemplo, SciELO Data, OSF, Zenodo), garantindo que o sistema gere um DOI ativo assim que o depósito for concluído.

2. Verifiquem o link DOI resultante, confirmando que redireciona corretamente para o conjunto de dados, com metadados completos e opção de download.

3. Atualizem a seção “Disponibilidade de dados” do manuscrito informando exatamente esse DOI funcional, sem aguardar qualquer liberação futura vinculada à publicação do artigo.

4. Incluam a referência completa do banco de dados na seção de “Referências” e citem-na no corpo do texto (preferencialmente em “Métodos”), seguindo o exemplo abaixo:

Andrade M. Estudo de genes em ratos albinos na América Latina. OSF [dataset], 2018, ASM0000v1. http://dx.doi.org/10.1590/0123-45620187214

Reproduzimos a diretriz da RESS para esse item:

“Os dados de pesquisa (bancos de dados, códigos, métodos e outros materiais utilizados e resultantes da pesquisa) idealmente devem ser depositados em repositórios de dados confiáveis como o repositório SciELO Data (lista disponível em https://wp.scielo.org/wp-content/uploads/Lista-de-Repositorios-Recomendados_pt.pdf), preferencialmente com um identificador persistente como o digital object identifier (DOI). O acesso a tais dados com o link para o repositório deverá ser informado na seção ‘Disponibilidade de dados’. Deve haver citação dos dados no texto, preferencialmente nos ‘Métodos’, e a referência completa deve ser incluída na seção de ‘Referências’.”

Dessa forma, garantimos conformidade com os requisitos de ciência aberta da RESS e evitamos dúvidas quanto ao acesso aos dados.

Observamos que, na seção “Disponibilidade de dados”, consta:

“O banco de dados e a descrição das variáveis estão disponíveis em: https://doi.org/10.48331/scielodata.UOZXWF.”

No entanto, este link não está acessível, retornando “página não encontrada”.

Recommendation: Minor revision

## Sixth round comments

Agradecemos a submissão do manuscrito RESS-2024-0899 intitulado “Fatores associados e tendência temporal da incapacidade física da hanseníase no Brasil: estudo de série temporal, 2014-2023” à Revista Epidemiologia e Serviços de Saúde: revista do SUS (RESS).

O título do manuscrito apresenta corretamente o delineamento como estudo de série temporal, porém, observamos que no resumo e na seção de métodos o estudo foi descrito como transversal.

Solicitamos que uniformizem a descrição do delineamento em todas as seções do manuscrito, adotando a classificação de estudo de série temporal.

Recommendation: Minor revision

